# Late-Onset Sepsis as a Risk Factor for Bronchopulmonary Dysplasia in Extremely Low Birth Weight Infants: A Nationwide Cohort Study

**DOI:** 10.1038/s41598-019-51617-8

**Published:** 2019-10-29

**Authors:** Euiseok Jung, Byong Sop Lee

**Affiliations:** Department of Pediatrics, University of Ulsan College of Medicine, Asan Medical Center, Seoul, Republic of Korea

**Keywords:** Neonatology, Paediatric research, Risk factors

## Abstract

This study aimed to determine the effect of late-onset sepsis (LOS) on the development of bronchopulmonary dysplasia (BPD) in extremely low birth weight (ELBW) infants. A prospective cohort study was performed using data collected from 64 centres registered in the Korean national registry. LOS was defined as a positive blood culture and antibiotics treatment after 72 hours of life and prior to 36 weeks postmenstrual age (PMA). Data on the causative organisms were collected and analysed for respiratory outcomes. Among the 1,434 ELBW infants who survived to 36 weeks PMA, 481 (34%) developed LOS caused by bacteria (n = 405), fungi (n = 28), or both (n = 48). The incidence of BPD was significantly associated with LOS in both the entire cohort and the propensity score-matched cohort. Two or more LOS episodes were a risk factor for BPD. The impact of multiple episodes of LOS on BPD was prominent in infants who received mechanical ventilation for two weeks or less. The estimated odds ratios for BPD and severe BPD were greater with fungal LOS than with bacterial LOS. In conclusion, LOS, particularly complicated by multiple episodes and/or fungi, was a risk factor for BPD in ELBW infants.

## Introduction

Inflammation in utero and during the postnatal period is one of the key underlying mechanisms in the development of bronchopulmonary dysplasia (BPD)^1^. The major prenatal factors that modulate foetal lung inflammation include maternal chorioamnionitis and antenatal corticosteroids, although their roles in the development of BPD remain controversial^2–4^. However, a consensus is being formed about the postnatal risk factors for BPD, which share a common mechanism that is characterised by the production of inflammatory cytokines^5^. These risk factors include respiratory distress syndrome (RDS), invasive mechanical ventilation (IMV), oxygen and sepsis^3,6^. Several treatment strategies to minimise lung inflammation have been attempted, but the incidence of BPD has remained grossly unchanged over the past two decades^7^.

Approximately 38% of extremely low birth weight (ELBW; those under 1 kg at birth) infants suffer from late-onset sepsis (LOS)^8^. The trend of LOS incidence roughly tracks in parallel with that of BPD in large cohort studies^9^. Thus, LOS could be a promising target for reducing the incidence of BPD in ELBW infants. Several retrospective studies have also reported an association between LOS and BPD in preterm infants^2,10,11^; however, it is difficult to ascertain the causative role of LOS in the development of BPD. There are several confounders that are linked to LOS and BPD, including prematurity and postnatal steroids. In addition, whether the lung injury is attributable to systemic or local inflammation caused by LOS, or to LOS-associated interventions, such as oxygen and mechanical ventilator therapy, remains unclear.

The severity of inflammation is a matter of interest for the risk assessment of BPD. There are limited data regarding the association between the specific characteristics of LOS and BPD. Among preterm infants who were subjected to neonatal sepsis, approximately 25% experienced two or more episodes of LOS^12^. However, no previous studies have focussed on the effect of repeated LOS episodes on BPD and its severity. In addition, no studies have thoroughly investigated the impact of different causative organisms on BPD.

The purpose of this study was to determine the causal relationship between LOS and BPD and to address the characteristics of LOS that increase the risk of BPD in ELBW infants by evaluating a nationwide cohort of preterm infants.

## Results

During the study period, 2,073 ELBW infants were registered in the Korean Neonatal Network (KNN) registry. Among them, 639 infants were excluded for death prior to 36 weeks postmenstrual age (PMA) (n = 544), major congenital anomalies (n = 44) and positive blood culture results ≤72 hours after birth (n = 51).

Of the 1,434 ELBW infants who fulfilled the inclusion criteria, 481 (33.5%) were diagnosed with LOS. The mean time at diagnosis of the first LOS episode was 27.3 (10–41) days after birth. LOS was caused by either bacteria (n = 405), fungi (n = 28), or both (n = 48). A total of 724 LOS episodes were reported among the 481 infants, and 36.4% (175/481) of the infants experienced multiple episodes, as follows: 2 (n = 120), 3 (n = 344), 4 (n = 49) and 5 (n = 2) episodes. For ELBW infant survivors who experienced bacterial sepsis, the rate of gram-positive and gram-negative bacteria was 84.1% and 15.9%, respectively. Coagulase-negative *Staphylococci* (CoNS) was the most common organism with an incidence of 40.3% (Table 1).

**Table 1:** Distribution of microorganisms associated with LOS episodes.

Microorganism	Number of Episodes	%
**Gram-positive organisms**	**540**	**73.1**
Coagulase-negative *Staphylococcus aureus*	292	40.3
Other *Staphylococcus*	91	12.6
Coagulase-positive *Staphylococcus aureus*	88	12.2
*Enterococcus*	45	6.2
Other *Streptococcus*	7	1.0
Group B *Streptococcus*	6	0.8
Others	11	1.5
**Gram-negative**	**102**	**13.8**
*Klebsiella*	26	3.6
*Serratia*	15	2.1
*Acinetobacter* species	14	1.9
*Enterobacter*	13	1.8
*Burkholderia* species	12	1.7
*Escherichia coli*	7	1.0
*Pseudomonas*	6	0.8
*Stenotrophomonas maltophilia*	4	0.6
*Proteus*	1	0.1
Others	2	0.3
**Fungi**	**82**	**11.3**
*Candida*	81	11.2
Others	1	0.1

Clinical characteristics and neonatal outcomes were compared between the infants with LOS (LOS group) and those free from LOS (no LOS group). The LOS group had a lower gestational age and birth weight and higher incidences of most neonatal morbidities than the control group. Regarding respiratory morbidities, the LOS group had a higher incidence of BPD, severe BPD, use of corticosteroids and home oxygen therapy as well as a longer duration of IMV support than the control group. The rate of LOS was lower in neonatal intensive care units (NICUs) with 40 or more beds. The associations between LOS and respiratory outcomes including BPD and longer hospital stays were identified with propensity score matching (Table 2).

**Table 2:** Characteristics of infants with and without LOS prior to 36 weeks PMA.

Characteristics	All infants			Propensity score-matched infants^*^				
	LOS (*n* = 481)	No LOS (*n* = 953)	*P*-value		LOS (*n* = 461)	No LOS (*n* = 461)	*P*-value	SMD
Gestational age (weeks)	25.9 ± 1.5	26.4 ± 1.6	<0.001		26.0 ± 1.5	26.1 ± 1.6	0.253^†^	0.065
Birth weight (g)	768.68 ± 143	801.4 ± 142.7	<0.001		773 ± 142.7	770 ± 149.4	0.738^†^	0.020
Small for gestational age, n (%)	63 (13.1)	143 (15)	0.331		60 (13)	63 (13.7)	0.847^‡^	0.019
Male, n (%)	234 (48.6)	428 (44.9)	0.180		221 (47.9)	206 (44.7)	0.351^‡^	0.065
Multiplicity, n (%)	162 (33.7)	274 (28.8)	0.055		151 (32.8)	142 (0.8)	0.559^‡^	0.042
Maternal hypertension	73 (15.2)	189 (19.8)	0.037		72 (15.6)	74 (16.1)	0.929^‡^	0.012
Maternal diabetes	27 (5.6)	60 (6.3)	0.609		27 (5.8)	29 (6.3)	0.892^‡^	0.018
Histologic chorioamnionitis, n (%)	162/392 (41.3)	353/829 (42.6)	0.678		157/380 (41.3)	159/376 (42.3)	>0.999^§^	0.029
PROM, n (%)	189/477 (39.6)	366/947 (38.6)	0.854		182/457 (39.8)	183/459 (39.9)	0.890^§^	0.054
Antenatal corticosteroids, n (%)	388 (80.7)	770 (80.8)	0.934		373 (80.9)	375 (81.3)	0.862^‡^	0.033
Caesarean section, n (%)	354 (73.6)	720 (75.6)	0.459		338 (73.3)	343 (74.4)	0.770^‡^	0.025
Apgar score at 1 minute, median (IQR)	4 (2–5)	4 (2–5)	0.116		4 (2–5)	4 (2–5)	0.826^†^	0.015
at 5 minutes, median (IQR)	6 (5–7)	6 (5–7)	0.010		6 (5–7)	6 (5–7)	0.621^†^	0.033
Resuscitation at birth, n (%)	467/475 (98.3)	932/948 (98.3)	0.996		449/457 (97.4)	449/457 (97.4)	>0.999^§^	0
RDS, n (%)	471 (97.9)	920 (96.5)	0.198		451 (97.8)	451 (97.8)	>0.999^‡^	0
Surfactant administration, n (%)	478 (99.4)	938 (98.4)	0.127		458 (99.4)	453 (98.3)	0.228^‡^	0.100
Treatment of PDA, n (%)	317 (65.9)	534 (56)	<0.001		301 (65.3)	306 (66.4)	0.779^‡^	0.023
NEC (stage ≥ 2), n (%)	74 (15.4)	69 (7.2)	<0.001		58 (12.6)	53 (11.5)	0.640^‡^	0.033
Systemic corticosteroids, n (%)	298 (62)	414 (43.4)	<0.001		280 (60.7)	271 (58.8)	0.507^‡^	0.040
Severe IVH, n (%)	82 (17)	105 (11)	0.002		72 (15.6)	70 (15.2)	0.923^‡^	0.012
ROP requiring laser, n (%)	153/442 (34.6)	194/850 (22.8)	<0.001		145/422 (34.4)	142/423 (33.6)	0.502^§^	0.018
Size of NICU^†^			<0.001				0.811^§^	0.039
low-volume, n (%)	72 (15.0)	150 (15.7)			72 (15.6)	70 (15.2)		
mid-volume, n (%)	318 (66.1)	505 (53.0)			305 (66.2)	300 (65.1)		
high-volume, n (%)	91 (18.9)	298 (31.3)			84 (18.2)	91 (19.7)		
BPD, n (%)	336 (69.9)	480 (50.4)	<0.001		317 (68.8)	272 (59)	0.001^‡^	
Severe BPD, n (%)	267 (55.5)	291 (30.5)	<0.001		251 (54.5)	177 (38.4)	<0.001^‡^	
Home oxygen therapy, n (%)	91 (18.9)	117 (12.3)	0.001		86 (18.7)	71 (15.4)	0.218^‡^	
IMV support duration, d (IQR)	48.3 (21–64.5)	29 (6–41.3)	<0.001		46.3 (20.5–62)	35.8 (13.5–48)	<0.001^†^	
Hospital stay, d (IQR)	118.1(89.5–131)	101.3 (79–114)	<0.001		116.6 (89–129)	109.1 (87–121)	0.005^†^	

Continuous variables are expressed as means ± standard deviations or IQR.

LOS, late-onset sepsis; SMD, standardised mean difference; PROM, premature rupture of membrane; RDS, respiratory distress syndrome; PDA, patent ductus arteriousus; NEC, necrotising enterocolitis; IVH, intraventricular haemorrhage; ROP, retinopathy of prematurity; NICU, neonatal intensive care unit; BPD, bronchopulmonary dysplasia IQR, interquartile range

^*^Infants were propensity score-matched on all baseline characteristics (except for BPD and hospital stay).

^†^The size of the NICU was classified according to the number of beds of each participating centre: 1–19 beds were classified as low-volume, 20–39 as mid-volume and 40 or more as high-volume.

^‡^Paired t-test, ^§^McNemar’s test and ^||^marginal homogeneity test were used after propensity score matching.

The ELBW infants who survived to 36 weeks PMA reported BPD and severe BPD incidences of 56.9% and 38.9%, respectively. Compared to the infants without BPD (no BPD group), those diagnosed with BPD (BPD group) had a lower gestational age and birth weight as well as a higher incidence of being male. The BPD group had a higher rate of most major neonatal morbidities than the no BPD group. In the multivariate analysis, lower birth weight, male sex, RDS, treatment for patent ductus arteriosus (PDA), systemic corticosteroids, severe intraventricular haemorrhage (IVH), retinopathy of prematurity (ROP) requiring laser photocoagulation and LOS were risk factors for BPD (Table 3).

**Table 3:** Characteristics of infants with a diagnosis of BPD versus no BPD at 36 weeks PMA.

	BPD (*n* = 816)	No BPD (*n* = 618)	*P*-value	Adjusted^*^ OR (95% CI)	*P*-value
Gestational age (weeks)	25.9 ± 1.6	26.7 ± 1.5	<0.001^†^	0.996 (0.907–1.095)	0.937
Birth weight (g)	763.5 ± 148.1	826 ± 129.2	<0.001^†^	0.997 (0.996–0.998)	<0.001
Male, n (%)	408 (50)	254 (41.1)	0.001^‡^	1.519 (1.194–1.933)	0.001
Multiplicity, n (%)	253 (31)	183 (29.6)	0.570^‡^		
Maternal hypertension, n (%)	146 (17.9)	116 (18.8)	0.670^‡^		
Histologic chorioamnionitis, n (%)	318/701 (45.4)	197/520 (37.9)	0.009^‡^	1.241 (0.948–1.626)	0.138
PROM, n (%)	324/808 (40.1)	231/616 (37.5)	0.319^‡^		
Antenatal corticosteroids, n (%)	647 (79.3)	511 (82.7)	0.264^‡^		
Caesarean section, n (%)	601 (73.7)	473 (76.5)	0.212^‡^		
RDS, n (%)	804 (98.5)	511 (82.7)	<0.001^‡^	1.832 (0.857–3.916)	0.118
Treatment of PDA, n (%)	549 (67.3)	302 (48.9)	<0.001^‡^	1.739 (1.358–2.228)	<0.001
NEC (stage ≥ 2), n (%)	96 (11.8)	47 (7.6)	0.009^‡^	1.275 (0.848–1.916)	0.243
Systemic corticosteroids, n (%)	500 (61.3)	212 (34.3)	<0.001^‡^	2.280 (1.778–2.923)	<0.001
Severe IVH, n (%)	145 (17.8)	42 (6.8)	<0.001^‡^	2.204 (1.483–3.276)	0.001
ROP requiring laser, n (%)	256/760 (33.7)	91/532 (17.1)	<0.001^‡^	1.392 (1.022–1.897)	0.036
LOS, n (%)	336 (41.2)	145 (23.5)	<0.001^‡^	1.581 (1.219–2.050)	0.001

Continuous variables are expressed as means ± standard deviations.

OR, odds ratio; CI, confidence interval; PROM, premature rupture of membrane.

^*^For gestational age, birth weight, sex, chorioamnionitis, RDS, the treatment of PDA, NEC stage ≥ 2, the administration of systemic corticosteroid, severe IVH, severe ROP, LOS and the size of the NICU.

^†^T-tests and ^‡^χ^2^ tests were used.

We investigated the characteristics of LOS episodes and their impact on respiratory outcomes. After adjusting for potential confounders in the multivariate analysis of the propensity score-matched cohort, two or more episodes of LOS were significantly associated with BPD and severe BPD. As the number of LOS episodes increased, the odds ratios for BPD and severe BPD also increased. The odds ratios for the risk of BPD and severe BPD were greater with fungal LOS than with bacterial LOS. Combined bacterial and fungal LOS had a higher odds ratio than bacterial or fungal LOS in the univariate analysis. However, the significance disappeared when adjusted for the confounding variables including the number of LOS episodes (Table 4).

**Table 4:** The characteristics of LOS and the risk of BPD in extremely low birth weight infants.

	Odds ratio (95% CI)					
	BPD			Severe BPD		
	Unadjusted	Adjusted for Baseline Covariates^*^	Propensity score-matched	Unadjusted	Adjusted for Baseline Covariates^*^	Propensity score-matched
All LOS	2.28 (1.81–2.88)	1.58 (1.22–2.05)	1.53 (1.17–2.01)	2.84 (2.26–3.56)	2.17 (1.69–2.79)	1.92 (1.48–2.49)
Single episode	1.64 (1.26–2.13)	1.25 (0.94–1.68)	1.13 (0.84–1.52)	2.22 (1.70–2.88)	1.87 (1.41–2.49)	1.52 (1.14–2.04)
Two episodes	3.75 (2.37–5.92)	2.36 (1.44–3.88)	2.55 (1.56–4.15)	3.30 (2.23–4.87)	2.19 (1.43–3.37)	2.30 (1.51–3.51)
Three or more episodes	9.85 (3.90–24.93)	5.17 (1.96–13.68)	5.84 (2.27–15.03)	10.24 (5.09–20.59)	6.48 (3.09–13.57)	6.78 (3.20–14.35)
Any bacterial	2.19 (1.73–2.77)	1.50 (1.15–1.95)^†^	1.42 (1.08–1.87)^†^	2.62 (2.08–3.29)	1.94 (1.51–2.51)^†^	1.70 (1.31–2.22)^†^
Any fungal	4.78 (2.50–9.13)	2.33 (1.15–4.74)^‡^	2.94 (1.51–5.70)^‡^	4.45 (2.66–7.45)	2.27 (1.28–4.03)^‡^	2.74 (1.59–4.73) ^‡^
Combined bacterial and fungal	8.75 (3.13–24.48)	1.51 (0.47–4.86)^§^	2.31 (0.74–7.14)^§^	4.97 (2.56–9.63)	0.94 (0.41–2.14)^§^	1.46 (0.65–3.27)^§^

^*^Baseline covariates were gestational age, birth weight, sex, chorioamnionitis, RDS, the treatment of PDA, NEC stage ≥ 2, the administration of systemic corticosteroids, severe IVH, severe ROP and the NICU level.

CI, confidence interval.

Odds ratios were further adjusted for ^†^fungal sepsis, ^‡^bacterial sepsis, or ^§^total number of sepsis episodes.

**Figure 1:**
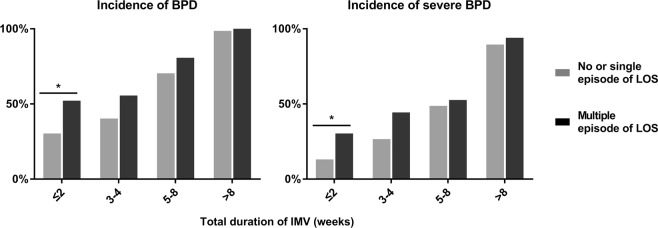
The number of LOS episodes and the incidence of BPD or severe BPD at each cumulative duration of IMV. Multiple LOS episodes of the infants who underwent IMV for ≤2 weeks were significantly associated with BPD or severe BPD. ^*^P < 0.05.

We investigated the association between multiple episodes of LOS and the respiratory outcomes in the subgroups categorised according to IMV duration. The impact of multiple LOS events on BPD and severe BPD was significant in the infants who received IMV for two weeks or less (Fig. 1).

The incidence of BPD did not differ between the infants with LOS caused by gram-positive bacteria (*p* = 0.954) and the infants with LOS caused by gram-negative bacteria (*p* = 0.954). No specific microorganisms, including CoNS (*p* = 0.452), increased the incidence of BPD relative to other bacteria.

## Discussion

Analyses of data from ELBW infants who survived to 36 weeks PMA revealed the following major findings. First, LOS was significantly associated with BPD and severe BPD, and the risk was further augmented by recurrent episodes of LOS. Second, relative to bacterial LOS, fungal LOS carried a greater risk of BPD. Third, there were no specific bacterial species that increased the risk of BPD relative to other bacteria. Our study had several methodological advantages over the aforementioned studies regarding the association between neonatal sepsis and BPD^2,3^. First, the data included were obtained from a large number of ELBW infants registered in a nationwide cohort. Second, detailed datasets on the episodes of neonatal sepsis in terms of diagnosis time and causative organisms were available. Third, the influence of many prenatal and postnatal variables that are potentially linked to both LOS and BPD were considered and adjusted for by propensity score matching. Notably, our results suggest that LOS acts as a risk factor for BPD, rather than simply being associated with it, in ELBW infants.

Despite a temporal association between the occurrence of LOS and the diagnosis of BPD, the causality between the two factors could not be clarified in practice. As the duration of IMV increases, preterm infants are subjected to both infection and superimposed ventilator-induced lung injury (VILI). As expected, the LOS group experienced a longer duration of IMV even in the propensity-matched cohort; the data on this finding have already been presented in a recent study using the same national cohort^13^. To determine the direct contribution of LOS to the development of BPD, the outcomes should be compared in study groups with minimal or at least a balanced risk of VILI. However, the design of such a study is not clinically feasible because the infants subjected to LOS inevitably require higher levels of respiratory and other types of supportive care that affect pulmonary outcomes. A few animal studies demonstrated that bacterial infection in the postnatal developing lung, either as a type of septicaemia or lipopolysaccharide injection, stimulates the inflammatory response and subsequent structural change of alveoli that is suggestive of BPD^14^.

To the best of our knowledge, no clinical studies have determined the dose-response relationship between the number of LOS episodes and the risk of BPD in preterm infants. In the present study, the odds ratios for BPD and severe BPD increased as the number of LOS episodes increased after adjusting for several confounding variables including chorioamnionitis, RDS, treatment of PDA and postnatal systemic corticosteroids. All of these parameters can alter systemic or local inflammatory responses and thereby affect pulmonary outcomes in preterm infants. For example, infection increases the ductal prostaglandin production and increases the risk of reopening or persistent PDA, which subsequently contributes to the worsening of lung functions due to the increase in pulmonary edema^15^. Considering the hypothesis of the ‘multiple hit’ model in the pathogenesis of BPD, our finding is not surprising and warrants the need for infection control as a key target for the prevention of BPD^5,16^. Notably, the association between recurrent LOS and BPD as well as severe BPD was most prominent in the subgroup of infants exposed to the shortest duration of IMV. This implies that recurrent LOS, if complicated during early postnatal days, has a significant impact on the development of BPD, irrespective of the VILI-dependent mechanism.

In the present study, the risk for BPD did not differ between the infants with LOS caused by CoNS and those with LOS caused by other bacteria. This is contrary to the results of a small retrospective study in which CoNS-related sepsis increased the risk for BPD compared to other bacteria; however, our results are in line with the results of another large cohort study^3,17^. The reason for this discrepancy remains unclear, but it may be due to differences in the study populations, the proportion of causative organisms and the treatment strategies employed. Because neonatal pro-inflammatory responses to CoNS are gestational age-dependent according to an *in vivo* study^18^, the differences in prematurity and the time of LOS occurrence might affect the pulmonary outcomes. In a cohort study from the Canadian Neonatal Network, the combined outcome of mortality and BPD was higher in infants with gram-negative bacteria than in those with gram-positive bacteria, but the difference was mainly attributable to the higher mortality in the group with gram-negative infections^10^.

In accordance with a large cohort study of very low birth weight infants^19^, fungal LOS was also associated with the risk of BPD, with a greater odds ratio than bacterial LOS. Compared to bacterial infection, fungal infection in rats induces a more severe lung injury characterised by capillary obstruction, interstitial haemorrhage and an elevated lung wet-to-dry ratio^20^. In a case-control study, candidemia was associated with the duration of IMV and previous bacteraemia, which are presumably the two most significant postnatal risk factors for BPD^21^. Because the incidence of isolated fungal infection was very limited in the present study, it remains unclear whether fungal LOS itself, mostly caused by systemic candidiasis, conferred a greater risk for BPD than bacterial LOS. Until now, there has been insufficient evidence that fungal prophylaxis reduces the risk of BPD, although a significant reduction of invasive candidiasis was reported in a recent randomised clinical trial^22^. Although the odds ratios were higher with combined bacterial and fungal LOS than with either bacterial or fungal LOS alone, the significance disappeared when adjusted for confounders including the number of LOS episodes. This suggests that repeated LOS episodes rather than the type of LOS (fungal or bacterial) increase the risk of BPD in preterm infants.

This study has several limitations. First, inherent to the retrospective study design, the treatment or prevention strategies for LOS and BPD may differ across the participating centres. Other treatment strategies that affect the risk of BPD, including fluid management, corticosteroid administration protocol and detailed information on ventilator care, were not available in our registry data. Second, because the main outcome of interest was BPD, the infants who died before 36 weeks PMA were excluded from the analysis. The mortality rate and the time of death following the occurrence of LOS vary depending on the causative organisms^23^. For example, the exclusion of infants who died from a severe single episode of LOS may result in selection bias in the final data analysis. Third, although adjusted for in the data analysis, the incidence of postnatal corticosteroid use was markedly higher in our study population, which consisted of survivors. This higher incidence of corticosteroid use might have influenced the incidence of LOS and BPD and mortality in an unpredictable direction^24,25^.

In conclusion, LOS, particularly if complicated by multiple episodes or fungal infection, increases the risk of BPD in ELBW infants. Our findings suggest that infection control should be a target for the prevention of BPD.

## Materials and Methods

### Study design

This cohort study used prospectively collected data from 64 NICUs participating in the KNN, a nationwide neonatal registry. Each participating centre recorded the study variables into the KNN data system according to the standardised KNN data manual^26^. For the present study, eligible infants included preterm infants with a birth weight <1,000 g who were born between 1 January 2013 and 31 December 2015. We excluded infants with major congenital anomalies, death prior to 36 weeks of postmenstrual age (PMA) and positive blood cultures ≤72 hours after birth.

### Data collection

Maternal data included hypertension, premature rupture of membrane, diabetes, histological chorioamnionitis and the use of antenatal corticosteroids. The data collected on the infants included sex, gestational age, birth weight, small for gestational age, multiple births, delivery mode, Apgar scores at 1 and 5 minutes, need for resuscitation at birth, the duration of IMV and morbidities, including RDS, PDA, necrotising enterocolitis (NEC), IVH, ROP, BPD and LOS. Data on the duration of hospital stay were also collected. According to the KNN manual of operation, LOS-causative microorganisms included 33 bacterial (*Achromobacter*, *Acinetobacter*, *Aeromonas*, *Alcaligenes*, *Bacteroides*, *Burkholderia*, *Campylobacter*, *Chryseobacterium*, *Citrobacter*, *Clostridium*, *Enterobacter*, *Enterococcus*, *Escherichia coli*, *Flavobacterium*, *Haemophilus*, *Klebsiella*, *Listeria*, *Moraxella*, *Neisseria*, *Pasteurella*, *Prevotella*, *Proteus*, *Providencia*, *Pseudomonas*, *Ralstonia*, *Salmonella*, *Serratia*, coagulase-positive *Staphylococcus aureus*, CoNS, other *Staphylococcus*, *Stenotrophomonas maltophilia*, group B *Streptococcus* and other *Streptococcus*) and 2 fungal (*Candida* and others) classifications.

### Definitions

Maternal hypertension included pre-existing hypertension and/or pregnancy-induced hypertension^27^. Antenatal corticosteroid administration was defined as either completion or incompletion of dexamethasone or betamethasone before delivery. BPD was defined as a requirement for supplemental oxygen or positive pressure support at 36 weeks PMA, with severe BPD defined according to the National Institutes of Health criteria^28^. An episode of LOS was defined as a positive blood culture result after 72 hours of life for one or more bacteria or fungi that were treated with appropriate antibiotics for five or more days and occurred prior to 36 weeks PMA^13,23^. A series of samples that were repeatedly positive for the same organism within seven days were considered part of the same episode^12^. If a different organism was cultured from a subsequent culture, this was considered an additional episode^23^. Multiple episodes of LOS were defined as two or more sepsis events regardless of the type of pathogen. The association of BPD with specific causative species of LOS was analysed using data from the first LOS episode of each infant. RDS was diagnosed based on typical chest x-ray findings and clinical symptoms requiring mechanical ventilator and/or non-invasive respiratory support after NICU admission. The treatment for PDA included medical treatment with ibuprofen or indomethacin and/or surgical ligation. NEC was confirmed using radiological or surgical findings, and only stage 2 or higher NEC was included for analysis according to Bell’s criteria^29^. Severe IVH was defined as grade III or IV according to Papile’s criteria^30^. The variation of care levels and treatment strategies between centres can influence the rates of LOS and BPD. However, data on the NICU level (i.e., level I, II and III) were not available in our KNN registry, and the provision of centre-identification data was not allowed according to KNN regulations^31^. We used the patient volume of the NICUs as an alternative for the level of NICU. The centres were categorised into three groups based on the number of NICU beds: 1–19 beds were classified as low-volume, 20–39 as mid-volume and 40 or more as high-volume.

### Ethical approval and informed consent

The registration of data in the KNN was approved by the institutional review board of each participating hospital, and all methods were performed in accordance with the relevant guidelines and regulations. Informed consent was obtained from the parents of each infant prior to participation in the KNN registry. This study was approved by the Asan Medical Center Instituitional Review Board.

### Statistical analysis

Continuous normally distributed variables were expressed as means and standard deviations or interquartile ranges and were compared with a t-test. Categorical variables were expressed as numbers and percentages and were compared with a χ^2^ test or Fisher’s exact test as appropriate. To analyse LOS as a risk factor of BPD, propensity score matching was performed including perinatal demographics, clinical characteristics and the number of beds in each participating NICU; LOS and no LOS matched groups were divided, and McNemar’s test, the marginal homogeneity test or a paired t-test were used. A multivariate logistic analysis was used to identify the factors associated with BPD, and the odds ratios were calculated by focussing on LOS. Using the previously prepared propensity score-matched data, the odds ratios of LOS were calculated as a risk factor of BPD and the number of beds and were compared. Statistical analyses were performed using SPSS version 23.0 (IBM Corp, Armonk, NY) and R version 3.1.2. Statistical significance was defined as *p* < 0.05.

## Data Availability

The Korean Neonatal Network (KNN) Publication Ethics Policy adheres to the following research data management and access guidelines: All information about patients and participating NICUs is confidential and is only available to individuals who have access for the purposes of the research activities permitted. Access is only allowed for the purpose of collecting data for the first time, and no access for any other purpose is allowed.
